# Bilaminar Chitosan Scaffold for Sellar Floor Repair in Transsphenoidal Surgery

**DOI:** 10.3389/fbioe.2020.00122

**Published:** 2020-02-25

**Authors:** Rodrigo Ramos-Zúñiga, Francisco López-González, Ivan Segura-Durán

**Affiliations:** ^1^Translational Neurosciences Institute, Department of Neurosciences, University Center of Health Sciences CUCS, Universidad de Guadalajara, Guadalajara, Mexico; ^2^Department of Neurosurgery, Hospital Civil de Guadalajara Fray Antonio Alcalde, Universidad de Guadalajara, Guadalajara, Mexico

**Keywords:** biomaterial, endoscopic, sellar floor reconstruction, chitosan, CSF leak, transsphenoidal

## Abstract

**Background:**

Endoscopic endonasal transsphenoidal surgery (EETS) is a standard technique used to approach sellar tumors. It is relatively safe, minimally invasive and carries a low risk of complications. However, one of the common complications reported with this technique is CSF leakage which causes morbidity, an increase in recovery time and hospital costs. This complication usually occurs from violation of the diaphragma sellae and a defect in the structures of the sellar floor or incomplete repair. In this article we report the first case with the use of a novel bilaminar chitosan scaffold which can be potentially used in the repair of the sellar floor, primarily aiming to the bony part of this structure.

**Case Presentation:**

After a personalized design employing a tissue engineering strategy, we reconstructed the sellar floor in a 65-year-old woman who had undergone EETS for a pituitary adenoma with progressive bilateral visual loss. To repair the bony defect of the sellar floor, we used a novel bilaminar chitosan scaffold. The patient had an unremarkable postoperative course with no evidence of CSF leak. The polymer was well tolerated without toxicity, infection or complications. After 2 years of follow up the patient remains neurologically intact, and in good endocrinological status.

**Conclusion:**

This is the first report of the use of this biomaterial and its biocompatibility in a clinical setting for the repair of the sellar floor during EETS. Our experience with chitosan bilaminar scaffold and in several preclinical studies in the literature have demonstrated good biocompatibility and effective bioengineered bone regeneration due to its excellent osteoconductive properties, this study pretends to be one landmark for further clinical research and larger case series with the use of this personalized tissue engineering materials in order to see they real efficacy to increase the surgeon armamentarium.

## Background

Transsphenoidal surgery has become a great tool for minimally invasive endoscopic resection of tumors in the anterior skull base, especially in the sellar region, there are, however complications largely reported from this approach, such as postoperative cerebrospinal fluid (CSF) rhinorrhea between others (Roca et al., 2018).

The frequency of postoperative CSF leaks depends on many factors as the technique used for reconstruction of the sellar floor and the skull base bony defect, and has been observed in as many as 5 to 75% of cases (Gardner et al., 2008a, b; Stippler et al., 2009; Greenfield et al., 2010). CSF leaks can lead to complications such as infection and pneumoencephalus, which may lead to further comorbidities, longer recovery times and increased hospital costs (Conger et al., 2018; Roca et al., 2018).

Different techniques and materials have been reported in the literature for reconstruction of these skull base defects including the use of fat grafts, autologous muscle, fascia lata, vascularized mucosal flaps, non-vascularized autografts, and different synthetic materials (Conger et al., 2018; Roca et al., 2018). They include bio-absorbable implants as collagen sponges (Kelly et al., 2001), dura mater substitute (Sandoval-Sánchez et al., 2012; Al-Asousi et al., 2017), fibrin glue, Polyethylene glycol (PEG) hydrogel dural sealant (Cosgrove et al., 2007; Burkett et al., 2011), polydioxanone plates and non-absorbable implants as porous polyethylene plates and titanium mesh (Al-Asousi et al., 2017).

Currently synthetic materials have become a great option to decrease and avoid donor-site morbidity and other complications related with the harvesting of autologous tissue (Al-Asousi et al., 2017).

The use of chitosan [poly-(β-1/4)-2-amino-2-deoxy-D-glucopyranose] has been described in several preclinical studies and tested in tissue bioengineering of bone (Zhao et al., 2002; Kim et al., 2004; Yoshida et al., 2004; Teng et al., 2008; Yuan et al., 2008; Liu et al., 2009; Xianmiao et al., 2009; Li et al., 2010; Mota et al., 2012; Pu et al., 2012; Zhang et al., 2012; Azevedo et al., 2014; Fan et al., 2014; Rodriguez-Vázquez et al., 2015; Vega-Ruiz et al., 2017) neural tissue (Simoes et al., 2011; Meyer et al., 2016; Ghasemi Hamidabadi et al., 2017; Nawrotek et al., 2017; Zhao et al., 2017) and soft tissue (Gobin et al., 2006; Paulo et al., 2009; Tchemtchoua et al., 2011; Udpa et al., 2013; Zou et al., 2017). Chitosan is a copolymer derived from the alkaline deacetylation of chitin and made of N-acetyl-D-glucosamine and D-glucosamine bonds and β bonds in which glucosamine is the main repeating unit in its structure (Rodriguez-Vázquez et al., 2015).

Observations made in studies of biosynthetic replacement of bone, the potential for reconstructive surgery was suggested due to its biocompatibility, as well as osteinductive and osteoconductive features (Zhao et al., 2002; Kim et al., 2004; Yoshida et al., 2004; Teng et al., 2008; Yuan et al., 2008; Liu et al., 2009; Xianmiao et al., 2009; Li et al., 2010; Mota et al., 2012; Pu et al., 2012; Zhang et al., 2012; Azevedo et al., 2014; Fan et al., 2014; Rodriguez-Vázquez et al., 2015; Vega-Ruiz et al., 2017). However, as of now there is a lack of clinical data demonstrating its usefulness and safety in the clinical setting (Vega-Ruiz et al., 2017).

Due to challenges frequently encountered in the repair of the sellar floor specifically the bony part, we intended to address this problem with a chitosan scaffold. This material has shown to provide useful characteristics facilitating bone regeneration (Zhao et al., 2002; Kim et al., 2004; Yoshida et al., 2004; Teng et al., 2008; Yuan et al., 2008; Liu et al., 2009; Xianmiao et al., 2009; Li et al., 2010; Mota et al., 2012; Pu et al., 2012; Zhang et al., 2012; Azevedo et al., 2014; Fan et al., 2014; Rodriguez-Vázquez et al., 2015; Vega-Ruiz et al., 2017), and scaffolding properties characterized by infiltration of fibroblasts, with subsequent deposits of organized collagen fibers, without evidence of infection or abnormal healing (Sandoval-Sánchez et al., 2012).

Our case describes the use of a novel bilaminar chitosan scaffold used for the repair of the sellar floor after endoscopic endonasal transsphenoidal surgery for a pituitary macroadenoma. Our use of such a bioactive membrane to repair the bony defect could potentially be useful for a strong and durable closure of the sellar floor, but further studies are needed to see its real efficacy, this case pretends to study the biocompatibility and performance of this material in a single patient and be a landmark for larger series.

## Bilaminar Chitosan Scaffold

**FIGURE 1:**
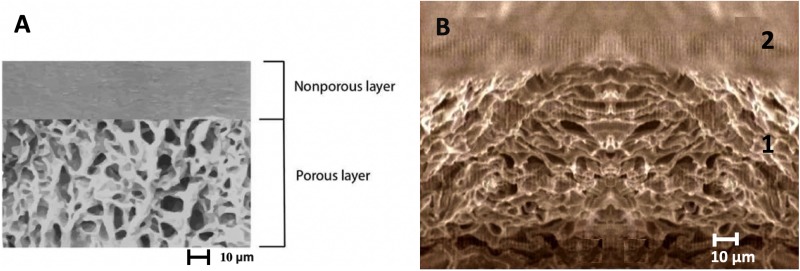
**(A)** Digital design that describe the biophysical structural characteristics of the chitosan scaffold in bilayer (Non-porous and porous) and the average pore diameter of 10 μm. **(B)** Electronic photomicrograph with evidence of the microstructure of the scaffold device substitute of dura mater conformed by two layers of chitosan. (1) Porous layer of 300 μm (2) Non-porous layer 100 μm. Observe the interface of the bi-layer that is structurally similar to the histological conformation of the dura mater, and has a homogeneous and regular pattern in the diameter of its pores, in contrast with the non-porous portion that has a smooth surface. This confers the capacity of an watertight behavior as a membrane on the one hand, in addition to forming a structural network for the development of fibroblasts of the receptor tissue to favor the orderly and structured development of the collagen in the regenerative process.

The bilaminar implant constitutes two types of different spatial structures: one of the membranes presents a flat-smooth structure, the other membrane has a tridimensional-porous structure. Each of the physical-chemical properties given to the membranes, was in anticipation of the biological effect needed in the effector tissue (Figures 1A,B).

The two types of membranes, synthetized for the construction of a bilaminar implant, were made with biomedical grade chitosan of medium molecular weight. It is characterized by 75–85% of deacetylation, and is available as a powder from Sigma Aldrich^®^, United States.

In the case of the membrane showing a flat-smooth structure, it was synthetized from a 2% chitosan solution made in diluted acetic acid acting as a solvent (Sigma Aldrich^®^, United States); In order to achieve a suitable solute, the mix was first set on a magnetic stirrer for 1 h. Then, the solution was brought into a sonicator at 28°C for 2 h to dissolve any air bubbles that had formed by stirring.

The other membrane (tridimensional-porous structure) was synthetized from a chitosan solution of 4% dissolved in diluted acetic acid (Sigma Aldrich^®^, United States);

The mix was set on a magnetic stirrer for 4 h, and thereafter the solution was brought to a sonicator at 28°C for 2 h,- again until all air bubbles formed by the stirrer were completely eliminated.

Once the solutions had been made, for the synthesis of the two membranes (the flat-smooth and the tridimensional-porous) both were set in comparable quantities (of ml/cm^2)^ in a Petri dish. In the case of the flat-smooth membrane, it was generated by drying under 98% of humidity loss and for the other (tridimensional-porous), a procedure of phase separating was thermically induced.

When both membranes were ready, they were assembled to create a sandwich structure. Consequently, the ensemble was put in a Petri dish and the cover lid was set up inverted. This construct was set up for drying for 24 h at room temperature and then it was precipitated in a solution of sodium hydroxide 1N, following the same protocol for each membrane separately before the construct was usable. Then we froze the mold at −196°C and then lyophilized it in a freeze dryer for 5 h (FTS Systems Inc., New York, NY, United States). The management of the implant with NaOH and the washing with distilled water result in a pH near to a neutral value.

The sterilization of the implant was performed with the use of ethylene oxide gas. We performed control tests to verify that the biomaterial structure was intact after the sterilization process. The reproducibility of the design and formulation of the chitosan scaffold has been systematized in the patent number: MX 358993 B in reference to the surgical procedure, synthesis and sterilization of the biomaterial. Nowadays is a standard procedure that combine the minimal invasive surgery and skull base surgery, in order to treat pituitary adenomas.

## Case Presentation

A 65 years old right-handed woman, came to neurosurgery for consultation due to progressive bilateral visual loss in her temporal fields. This had occurred over 10 months, and 2 weeks prior to her admission she reported sudden loss of consciousness, prompting her admission to the hospital. On examination, she was alert and oriented x 3, she had a normal cranial nerve examination except for decrease visual acuity (20/200 in her left eye, 20/80 in her right eye), bitemporal hemianopia and mild atrophy of the optic disk in the left eye. Gait, motor and sensory examination was normal.

**FIGURE 2:**
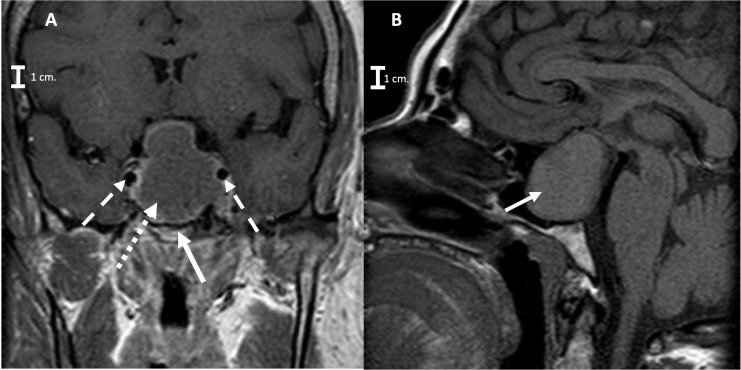
**(A)** Preoperative coronal Brain MRI T1 signals respectively, showing the pituitary macroadenoma (arrow with short dashed line) with suprasellar extension and selllar floor erosion (arrow with continues line), without encasement of the carotids (arrows with long dashed line pointing both carotids). **(B)** Sagittal brain MRI T1 signals respectively (arrow with continues line is pointing to the Macroadenoma).

**FIGURE 3:**
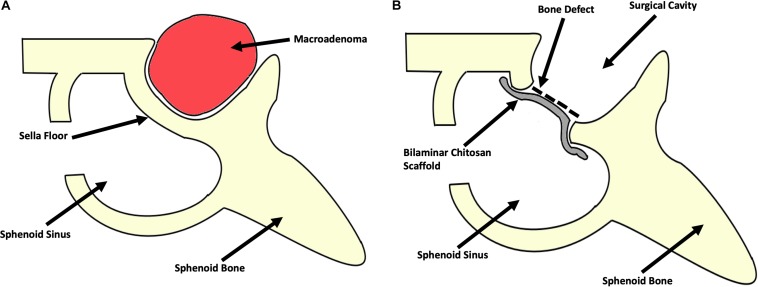
Schematic representation of the surgical procedure. **(A)** Preoperative representation of the surgical corridor and the anatomical location of the macroadenoma. **(B)** Postoperative representation of the surgical cavity after gross total resection of the macroadenoma and the reconstruction of the sellar floor bony defect with the bilaminar chitosan scaffold (due to the easy molding of this scaffold, it was possible to cover the entire shape of the bony defect).

**FIGURE 4:**
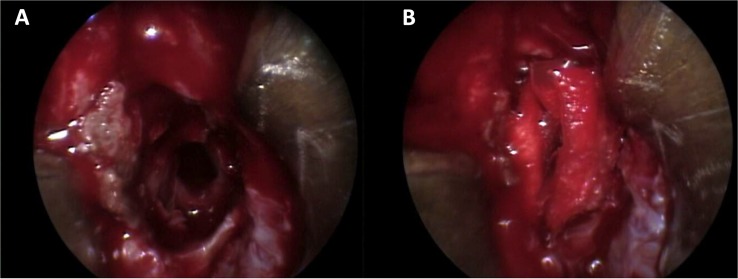
Transoperative images of the implanting of the bilaminar chitosan scaffold in the sellar floor defect, **(A)** image of the defect before the setting of the scaffold, **(B)** image of the sellar defect after the setting of the scaffold.

Laboratory studies showed a LH at 0.22 IU/L (reference value in Postmenopausal females 15.0–62.0 mIU/mL)and prolactin at 53 ng/mL (reference value in non-pregnant females 2–29 ng/mL)0.7. A contrast enhanced brain MRI was obtained (Figures 2A,B) and revealed a sellar lesion which was hypointense in T1 but hyperintense in T2 sequences with enhancement of the periphery. The lesion extended into the sphenoid sinus and parasellar space without encasement of the carotids and into the suprasellar cistern abutting the optic chiasm. The patient underwent endoscopic endonasal transsphenoidal surgery for resection of the sellar lesion (Figures 3A,B). Intraoperatively, the lesion appeared reddish in color and it was of soft consistency. Moderately bleeding was encountered during resection and a sample was taken for pathology. At the end of the tumor removal, the scaffold was implanted to close the bone defect in the sphenoid sinus (Figures 4A,B). Due to the fact that the graft could be molded into shape, it was easily set and allowed to cover the entire size of the defect. A standard fat graft was then placed in the sphenoid sinus covering the outer membrane of the chitosan graft. Finally, fibrin sealant was used, and a nasal packing was inserted in both nostrils.

**FIGURE 5:**
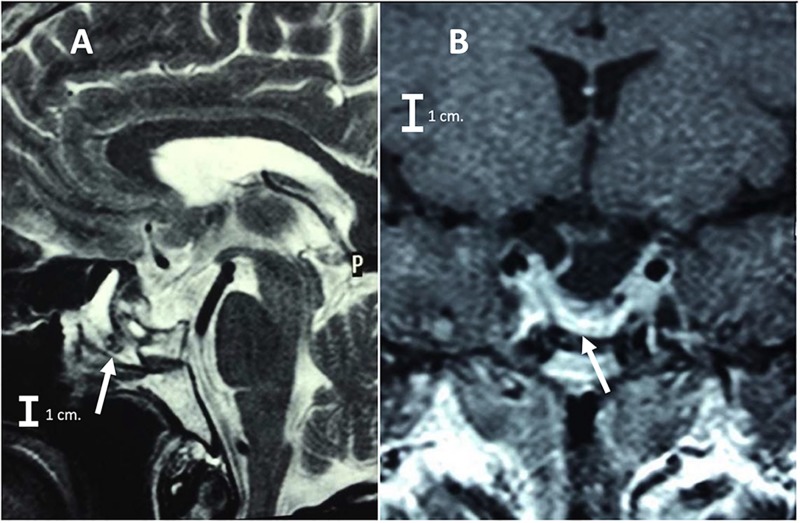
**(A)** Postoperative coronal brain MRI T1 signal respectively, showing the gross total resection of the Macroadenoma and the reconstruction of the sellar floor (arrow) with the bilaminar chitosan scaffold, **(B)** sagittal brain MRI T2 signal (arrow showing also the reconstruction of the sellar floor with the bilaminar chitosan scaffold).

The patient had an unremarkable postoperative period and after a few days the patient was discharged without evidence of CSF leak or complications. After one month the patient showed complete recovery of her visual acuity and visual fields. At follow up, the patient underwent a postoperative brain MRI (Figures 5A,B) illustrating gross total resection and good closure of the sellar floor. There were no signs of rejection or inflammation in the area where the chitosan scaffold was implanted.

## Discussion

One of the main challenges of transsphenoidal surgery is a potential CSF leak and a durable reconstruction of the sellar defect. The CSF leaks can derive from the surgical procedure or from primary erosion of the sellar floor which can be encountered in various tumors that affect this region and it remains a matter of debate of how to seal these off in the best way (Gardner et al., 2008a, b; Stippler et al., 2009; Greenfield et al., 2010; Conger et al., 2018).

In this article we present the first biocompatibility results of using a novel bilaminar chitosan scaffold construct for repair of the sellar floor primarily aiming to the bony defect, in endonasal endoscopic transsphenoidal surgery.

We consider chitosan a very suitable material, as the potential for bone regeneration and its physical-chemical characteristics have been already established and reported in the literature. However, there is a lack of data for its use in the clinical setting and the description of its biocompatibility in human tissues is also poor.

The inclusion of this patient with a macroadenoma to this clinical trial was performed, due to the larger surgical corridor needed for the resection of the voluminous mass in comparison with smaller lesions, the requirement for a larger bony defect in the sellar floor in order to extend the access of the resection, increase the complexity of the reconstruction of this structure and also rise the concern for a postoperative CSF leak. Also, this single patient was selected for an evaluation in terms of biocompatibility, toxicity, and the long-time biological behavior of the biomaterial before continuing to a larger series of patients. At this time, we are planning for a bigger clinical trial with the inclusion of more patients.

For that reason, we intended to show in this study that a bilaminar chitosan scaffold is a very appealing option for the bony part of sellar floor reconstruction as its use carries the advantage of chitosan being osteconductive. A bioengineered scaffolding with good mechanical properties could potentially allow a customized repair of the bone defects after surgery.

The use of chitosan was selected over other materials, due to its biodegradability, low cost, versatile molding and non-toxic properties reported in the literature, in applications such skin regeneration, tissue engineering and regenerative medicine (Sandoval-Sánchez et al., 2012; Rodriguez-Vázquez et al., 2015; Vega-Ruiz et al., 2017). The main concerns for complications with the use of this scaffoldings is the low risk for local inflammatory reactions and rejection of the implant.

The patient was discharged with oral pain medication, antibiotics, endocrinological follow up and instructed postoperatively to return to our hospital facilities at any symptom concerning of CSF leak, infection, local or systemic inflammatory response and was also followed up with a postoperative MRI and laboratory studies that included but are not limited to cell blood counts (CBC), acute phase reactants and blood chemistries. This case has been followed up for more than 3 years, without postoperative symptoms or complications, abnormalities in the laboratory or in imaging studies.

In our report we describe a good malleability of the scaffold during its intraoperative use. Advantageous practical feature are its moldable nature, the fact that it can be easily cut and adapted to the patient′s anatomical needs, allowing the surgeon to be very flexible during the surgical procedure.

The appropriate tolerance and biocompatibility between the implant and the recipient tissue were evaluated, and there was not evidence of local or systemic inflammatory reaction, toxicity, infection, or severe scar reaction or fibrosis. (Acute phase reactants and CBC were within the normal range. At the local level the postoperative MRI also showed a good reconstruction and sealing of the sellar floor without signs of rejection or inflammatory response per imaging). Accomplishing the surgical objective of reconstruction of the sellar floor.

**FIGURE 6:**
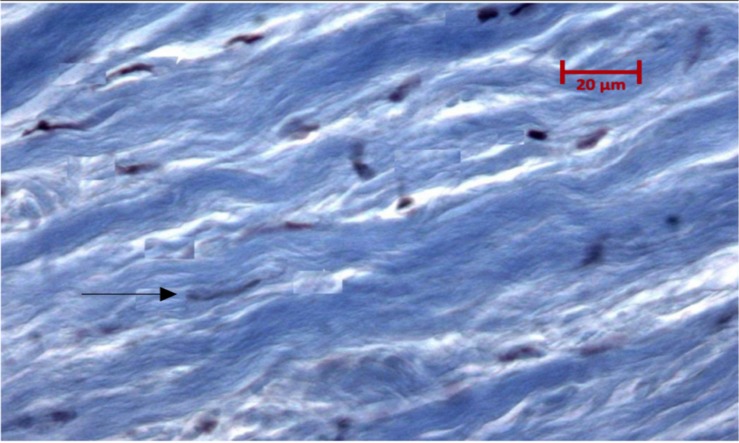
Photomicrograph with Masson trichrome stain 180 days after the colocation of scaffold in the preclinical experimental process in which an organized structure of fibroblast and collagen growth (arrow) is demonstrated without evidence of fibrosis or acute or chronic inflammatory reaction in the scaffold from which the dura mater has regenerated.

In the preclinical study, from our research group (Sandoval-Sánchez et al., 2012). The experimental model was performed in two phases: An *in vitro* phase were the bilayer chitosan scaffold was evaluated for pore size, thickness, water absorption capacity, tensile strength, strain, and toughness. In the second *in vivo* phase 27 durectomized New Zealand rabbits were randomly assigned into three duraplasty groups with autologous dura, collagen matrix or bilayer chitosan scaffold. Histology response to regeneration was evaluated through hematoxylin and eosin stain and Masson trichrome stain after the colocation of the implant (Figure 6; Sandoval-Sánchez et al., 2012).

We concluded that the biomaterial was biocompatible as in the *in vivo* phase there were no cases with CSF fistula, fever, or infection. In the histologic evaluation after 180 days we found an organized structure of fibroblast and collagen growth without evidence of fibrosis or acute or chronic inflammatory reaction in the scaffold from which the dura mater has regenerated. The scaffold did not compress neural tissue and conserved a watertight dural closure, these findings make the bilayer chitosan scaffold a suitable and biocompatible option for reconstruction of the sella floor.

With this study we wish to create a landmark for further testing of this biomaterial as we want to advance tissue engineering in neurosurgical procedures.

Further research is also needed to establish the biocompatibility in larger series, and to assess any potential side effects of chitosan in the clinical setting and to prove its usefulness and advantage compared with other materials already used in the clinic (Macías-Reyes et al., 2012; Sandoval-Sánchez et al., 2012; Rodriguez-Vázquez et al., 2015; Vega-Ruiz et al., 2017).

To this end it is necessary to conduct a comprehensive study of a series of such patients and to also evaluate the long-term efficacy of this therapeutic option, as well as its price tag, when compared with other materials.

## Conclusion

A novel chitosan scaffold was created and designed by bioengineering methods for tissue repair with the concept of reconstruction of the bony part of the sellar floor in mind.

In this single case report to elucidate the biocompatibility and safety from an observation with more than 3 years of follow up, we show that the chitosan scaffold demonstrated good biocompatibility, without any observed toxicity or inflammatory reaction which was feared as a host response against the implanted biopolymer.

We wanted to verify the suitability of the scaffold in a single case with a long prospective follow up before continuing to larger series and to stablish a landmark for further clinical studies. We theorize that this bilaminar scaffold could act not only as a mechanical barrier but also as a bioactive implant which is suitable for surgical repair of bony defects due to its permissive nature in bone regeneration as reported in several preclinical studies, further research is needed to demonstrate the real efficacy of this bioengineering scaffolds in the clinical settings.

## Data Availability Statement

The datasets generated for this study are available on request to the corresponding author.

## Ethics Statement

Bioethics Committee Approval: Register O63-2014. Center of Health. University of Guadalajara. All the procedures performed in this report were in accordance with the ethical standards of the institutional and/or national research committee and with the 1964 Helsinki Declaration and its later amendments or comparable ethical standards. Informed Consent for Participation: Informed consent was obtained from all individuals participants included in the study. Informed Consent for Publication: Informed consent was obtained from the participant for the publication of this case report and any potentially-identifying information/images. International Clinical Trial Register: NCT03280849.

## Author Contributions

RR-Z and FL-G contributed to the design and implementation of the research, to the analysis of the results, data acquisition and to the writing of the manuscript, revision for intellectual content, gave final approval of the version to be published, and agreed to be accountable for all aspects of the work in ensuring that questions related to the accuracy or integrity of any part of the work are appropriately investigated and resolved. IS-D contributed to the analysis of the results, data acquisition, and to the writing of the manuscript.

## Conflict of Interest

The authors declare that the research was conducted in the absence of any commercial or financial relationships that could be construed as a potential conflict of interest.
